# Supplementary material for: Are carbon emissions trading and green financial instruments synergistic? -Comprehensive quantitative research based on content analysis
Source: PLoS One. 2024 Mar 7;19(3):e0298601. doi: 10.1371/journal.pone.0298601 (PMC10919601; doi:10.1371/journal.pone.0298601)
Supplement: S1 Data — (ZIP) [file pone.0298601.s001.zip › Data/地级市Z_F工作报告.zip!/╣π╬≈/░┘╔1⁄2╩╨/└┤╫╘╣1⁄2╓┌║┼í╛┬φ┐╦╩2╛▌═°í┐_2015.docx]

一、2014年工作回顾

过去一年，困难和挑战前所未有，面对市场价格下滑、支柱产业受挫、财政增长困难、融资难度加大等多重困难叠加的复杂形势，在市委的正确领导下，在市人大、市政协的监督支持下，我们牢牢把握稳中求进工作总基调和改革创新、扩大开放工作主线，坚持“四个优先”，深入实施“四大攻坚战”，主动作为，开拓进取，奋力攻坚，全市经济社会发展取得了重大成绩。

——成功举办纪念邓小平同志诞辰110周年暨百色起义85周年系列活动；

——百色生态型铝产业区域电网顺利组建，一期工程全面开工；

——百色至靖西、靖西至那坡两条高速公路正式通车；

——百色至上海航线正式开通；

——建成百色园博园，成功举办第五届广西（百色）园林园艺博览会；

——广西百色干部学院开工建设；

——中国－东盟农产品交易中心获国家商务部批复建设；

——成功创建“国家卫生城市”；

——“百万亩芒果产业”工程和“田七回家”工程顺利推进；

——成功举办第七届中国－东盟（百色）现代农业展示交易会。

（一）经济运行稳中有进。

全市生产总值917.9亿元，增长8.4%，增速排在全区第5位，比上年提升5位。财政收入完成108.7亿元，增长1%。固定资产投资完成895.2亿元，增长11.6%。规模以上工业增加值完成386.3亿元，增长9.4%。社会消费品零售总额201亿元，增长12.6%。居民消费价格指数上涨2.3%，控制在5%的目标内。城镇新增就业2.1万人，城镇登记失业率控制在4%的目标内。外贸进出口总额7.3亿美元，增长21.8%。

（二）人民生活持续改善。

扶贫攻坚十大工程扎实推进。建设通村道路1618公里，实施危房改造2.75万户，饮水安全工程898个，贫困家庭子女就读普通本科高校、高等和中等职业院校全部实现应补尽补。全面完成精准扶贫建档立卡工作。新型扶贫生态移民安置社区建设稳步推进，搬迁3700户1.6万人。预计年内减贫16.5万人。公共财政用于民生领域支出212.8亿元，增长14.2%，占公共财政预算支出81.5%。6个农民工创业园建设取得新进展。农村劳动力转移就业新增5.5万人次。城乡低保、五保对象、0～3公里边民和双拥优抚对象补助标准进一步提高。五项社会保险参保总人数113.9万人次，增长6.6%。新开工保障性住房9412套，基本建成9719套，分配入住7857套。十项为民办实事工程全面完成。城镇居民人均可支配收入23496元，增长9.5%；农民人均纯收入6166元，增长14%；城乡居民收入增速分别比GDP高1.1和5.6个百分点，城乡收入比继续缩小。

（三）结构调整稳步推进。

特色农业发展势头良好。“南菜北运”基地、现代特色农业（核心）示范区、“双高”糖料蔗基地、规模化畜禽牧渔特色养殖基地建设扎实推进。全市芒果和田七种植面积分别达到66 万亩和1.9万亩，肉鸡出栏和水产品产量增幅均排全区第1位。成功在北京、上海、香港等地举办“百色芒果行销全国”系列推介活动。 “百色芒果”、“百色番茄”获国家农业部农产品地理标志保护登记认证，“乐业雅长铁皮石斛”成为国家地理标志保护产品，“百林鸡”商标通过国家工商总局注册。新增农民专业合作社454家，家庭农场90个，新认定市级农业产业化重点龙头企业18家。病险水库除险加固、中小河流治理、农田水利等工程建设全面提速。《百色水库灌区规划》通过水利部专家评审。

工业结构调整加快。百色生态型铝产业区域电网一期工程全面开工建设。广西信发、广西苏源、百矿集团等重点煤电铝一体化项目进展顺利，部分实现竣工投产。铝土矿赤泥综合利用提前1年实现国家批复的目标任务。铝材加工由建筑型材逐步向铝板带箔、铝镁合金线、汽车铝轮毂等高附加值的铝深加工产品发展，铝及配套产业工业总产值突破600亿元。全市电解铝产量下降22%，但铝材产量达166万吨，增长22.9%，铝及配套产业增加值增长13%。碳酸钙、铝锰基等新材料产业加快发展，机械制造、生物制药、电子信息等新兴产业比重上升。

服务业加快发展。第三产业增速比上年提高0.6个百分点。凌云县、乐业县、田阳县、右江区纳入自治区规划的巴马长寿养生国际旅游区，百色起义纪念园、乐业天坑群、靖西大峡谷景区创5A工程、特色旅游名县创建工作扎实推进。全市接待游客数量、旅游综合收入同比分别增长18.9%和27.9%。中心城区“十大市场工程”、恒宁城市广场等大型城市综合体和各县（区）一批商贸物流重大项目建设取得新进展。百色一号专列稳定运营，壮乡河谷、古鼎香等农产品综合批发市场冷链仓储项目开工建设。现代物流业、金融服务业、电子商务等新业态发展态势良好。公路客运周转量增长14.8%，货物周转量增长10.6%。

（四）发展质量稳中向好。

新增年销售收入超亿元企业13家、规模以上企业30家、微型企业670家。民生领域投资增长25%，高于固定资产投资增速13.4个百分点。房产经营服务投资和商务服务投资分别增长1.78倍和5.59倍。公共财政预算支出增幅比预算收入高4.3个百分点，非税收入占比下降1.3个百分点。八大高耗能行业增加值比重下降2.3个百分点。田东石化工业园区升级为国家级生态工业园区，是全区唯一获此殊荣的园区。万元GDP能耗下降6%，提前1年完成“十二五”目标任务。化学需氧量、氨氮、氮氧化物、二氧化硫排放量四项控制指标同比下降。全市环境空气质量和主要河流水质总体保持良好，城区空气质量优良天数达349天，饮用水源地水质达标率100%。森林覆盖率达67.12%，植树造林面积连续4年排全区第1位。荣获“广西森林城市”称号。“美丽百色·清洁乡村”工程取得良好成效，成功创建一批自治区级和市级生态村，乡村环境和面貌明显改善。

（五）互联互通取得突破。

全市9个县（区）通达高速公路，通达率75%，高于全区2个百分点。云桂高铁和平果至马山、崇左至靖西高速公路加快建设，南昆铁路二线百色段和河池至百色、靖西至龙邦高速公路开工建设，百色至乐业、田林至西林高速公路项目前期工作有序推进。建制村沥青（水泥）路通畅率达80.9%，85%行政村开通客运专线。百色机场盲降系统通过专家论证，百色巴马机场迁建选址完成前期工作。百色港大旺作业区、田阳头塘、二塘作业区及田东港祥周作业区等黄金水道重点工程取得新进展。

（六）新型城镇化快速发展。

百东新区建设、右江—田阳一体化进程加快。中心城区龙景核心区路网基本建成贯通，龙旺大桥、城南环路建成通车，环岛一路、环岛二路建设和半岛公园改造提升、右江沿岸综合整治工程进展顺利。各县（区）城镇化建设取得新成绩。平果县和靖西县撤县设市顺利推进。右江河谷城镇带建设步伐加快。荣获全区第九届城市市容环境综合整治“南珠杯”竞赛特等奖。靖西县、德保县、田东县、乐业县荣获“广西园林城市”称号，创建“广西园林城市”数量领先全区。全市建设领域投资完成290.6亿元，增长37.4%。城镇化率达41.5%，提高2个百分点。

（七）深化改革激发活力。

实施改革攻坚战，推进8大方面33个重点事项改革。全区深化农村金融改革在我市全面启动铺开。农村土地承包经营权及宅基地、住房确权登记颁证试点工作有序开展，成立全市第2家县级农村产权交易中心。集体林权制度改革取得新成效，新增林权抵押贷款4.4亿元。农业科技园区、华侨农场体制改革和百色饭店、广西建华机械有限公司等一批国有企业改革稳步推进。完成工商注册资本登记制度改革任务，全市新登记公司制企业和注册资本分别增长1.95倍和4.4倍。理顺城区社区卫生服务中心管理体制，并完成移交下放。投融资体制改革成效显著。交通银行、柳州银行在百色开设分行。沿边金融综合改革试验区建设启动，跨境人民币结算达14.4亿元，增长1.2倍。资本市场实现多种形式融资，主要融资平台通过金融市场融资105.5亿元，增长2.1倍。城市管理综合执法体制改革顺利推进。

（八）开放合作深化拓展。

抓住广西实施“双核驱动”发展战略机遇，主动融入21世纪海上丝绸之路、珠江－西江经济带，积极推进左右江革命老区振兴规划。参与推进并出台“南北钦防玉崇百”七市区域一体化发展规划。与广州市的扶贫开发以及上海交大、中山大学的产学研合作取得新进展。引进央企参与百矿集团煤电铝一体化等重大项目建设，打通了香港、深圳、上海等地项目和资金进入百色的新渠道。与云南文山州共建产业园区取得实质性进展。中国—东盟农产品自由贸易示范园区建设启动。沿边开发开放试验区管委会正式成立，边境经济合作区申报工作启动，龙邦口岸升格为国际性口岸以及平孟国家一类口岸开放工作加快推进。

（九）社会事业
